# The Role of Phospholipase Activity of Peroxiredoxin 6 in Its Transmembrane Transport and Protective Properties

**DOI:** 10.3390/ijms232315265

**Published:** 2022-12-03

**Authors:** Mars G. Sharapov, Ruslan G. Goncharov, Svetlana B. Parfenyuk, Olga V. Glushkova, Vladimir I. Novoselov

**Affiliations:** Institute of Cell Biophysics of the Russian Academy of Sciences, FRC PSCBR RAS, 142290 Pushchino, Russia

**Keywords:** peroxiredoxin 6 (Prdx6), phospholipase A2, transmembrane transport of proteins, oxidative stress, ionizing radiation, radioprotection

## Abstract

Peroxiredoxin 6 (Prdx6) is a multifunctional eukaryotic antioxidant enzyme. Mammalian Prdx6 possesses peroxidase activity against a wide range of organic and inorganic hydroperoxides, as well as exhibits phospholipase A2 (aiPLA2) activity, which plays an important role in the reduction of oxidized phospholipids and cell membrane remodeling. Exogenous Prdx6 has recently been shown to be able to penetrate inside the cell. We hypothesized that this entry may be due to the phospholipase activity of Prdx6. Experiments using exogenous Prdx6 in three cell lines (3T3, A549, RAW 264.7) demonstrated that it is the phospholipase activity that promotes its penetration into the cell. Overoxidation of Prdx6 led to a suppression of the peroxidase activity and a 3-to-4-fold growth of aiPLA2, which enhanced the efficiency of its transmembrane transport into the cells by up to 15 times. A mutant form of Prdx6-S32A with an inactivated phospholipase center turned out to be unable to enter the cells in both the reduced and oxidized state of the peroxidase active center. Previously, we have shown that exogenous Prdx6 has a significant radioprotective action. However, the role of phospholipase activity in the radioprotective effects of Prdx6 remained unstudied. Trials with the mutant Prdx6-S32A form, with the use of a total irradiation model in mice, showed a nearly 50% reduction of the radioprotective effect upon aiPLA2 loss. Such a significant decrease in the radioprotective action may be due to the inability of Prdx6-S32A to penetrate animal cells, which prevents its reduction by the natural intracellular reducing agent glutathione S-transferase (πGST) and lowers the efficiency of elimination of peroxides formed from the effect of ionizing radiation. Thus, phospholipase activity may play an important role in the reduction of oxidized Prdx6 and manifestation of its antioxidant properties.

## 1. Introduction

Peroxiredoxin 6 (Prdx6) is an important eukaryotic antioxidant enzyme belonging to an ancient group of thioredoxin-like peroxidases. Prdx6 is a moonlighting protein since it exhibits, along with the peroxidase activity, properties of Ca^2+^-independent phospholipase A2 (aiPLA2–acidic Ca^2+^-independent PLA2) and acyl-transferase (LPCAT) [1,2]. The most important feature of Prdx6 resides in its ability to reduce a wide range of organic and inorganic peroxides in the cell [3]. Unlike other members of the peroxiredoxins family, Prdx6 is able to reduce not only H_2_O_2_, alkyl hydroperoxides (ROOH), and peroxynitrite (ONOO^−^), but also peroxides of phospholipids (PLOOH) [4]. It should be mentioned that under normal conditions the intracellular concentration of peroxides (H_2_O_2_) basically does not exceed 1–5 μM, reaching 150 μM upon development of pathological processes, whereas its levels above 200 μM result in cell death [5,6]. Interestingly, Prdx6 exhibits a maximum peroxidase activity particularly in these micromolar concentrations of hydroperoxides since the Michaelis constant values (app. Km) for these substrates fall in the range of 100–200 μM [7]. Prdx6 is abundant in epithelial tissues where it performs an important antioxidant function. For example, in the lung tissue, which is particularly susceptible to oxidative stress, Prdx6 provides up to 70% antioxidant protection [8]. Prdx6 has been shown to be present in all the areas of the respiratory tract [9]. This protein reduces phospholipid hydroperoxides and takes part in the metabolism of lung surfactant through its phospholipase activity [2]. Peroxidase activity of Prdx6 promotes reduction of oxidized fatty acids (to corresponding alcohols), while aiPLA2 activity ensures hydrolysis of the phospholipid acyl group in the 2nd position (sn-2). The resulting lysophosphatidylcholine (LPC) can be reacylated due to the acyl transferase activity (LPCAT) of Prdx6 [2]. Deacylation and reacylation reactions catalyzed by Prdx6 take place without the release of LPC from the enzyme [2]. Thus, because of its catalytic properties, Prdx6 plays an important role in the regulation of the redox homeostasis of the cell and maintenance of the cell membrane integrity [7].

**Figure 1:**
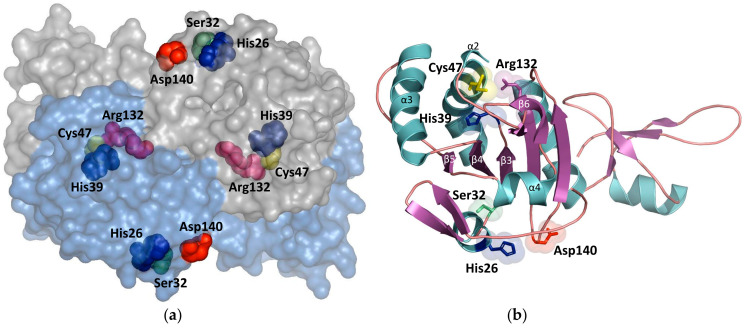
Human Prdx6 catalytic centers: peroxidase (His39, Cys47, Arg132) and phospholipase–aiPLA2 (His26, Ser32, Asp140). (**a**) A “surface” model of Prdx6 with indication of positions of active centers in the dimeric form of protein. Prdx6 is crystallized as a homodimer (PDB ID: 5B6M), and the aiPLA2 center is constantly available for substrates, whereas the peroxidase center is closed. During the catalytic cycle, Prdx6 undergoes heterodimerization with πGST, which, with the involvement of glutathione, reduces the oxidized Cys47 (C_P_-SOH). (**b**) Secondary structure of Prdx6 with indication of amino acids included in the active centers. Secondary structure elements (α2-4; β3-6) forming the thioredoxin fold are marked. Visualization in PyMol v.0.99.

Peroxiredoxins have an evolutionary ancient thioredoxin fold represented by three α-helices and four β-strands (Figure 1b), which is typical for many thiol-containing oxidoreductases [10]. In mammals, six types of peroxiredoxins have been identified, which are divided according to the number of cysteine residues in the active center, and the mechanisms of catalysis, into: 1-Cys (Prdx6), typical 2-Cys (Prdx1-4), and atypical 2-Cys (Prdx5). The peroxidase activity of all peroxiredoxins is determined by the presence of a conserved cysteine residue C_P_ (peroxidatic cysteine) in the N-terminal region of the polypeptide chain. Prdx6 is the only representative of 1-Cys peroxiredoxins. 2-Cys peroxiredoxins (Prdx1-5) carry an additional “reducing” cysteine residue, C_R_ (resolving cysteine), in the C-terminal region of the protein. Amino acids forming the peroxidase catalytic center are highly conserved for all peroxiredoxins [11]. The peroxidase active center of Prdx6, including the His39, Cys47, and Arg132 triad (Figure 1), is located in a globule pocket with a diameter of approximately 4 Å [12]. Upon reduction of peroxides, the thiol group of the Prdx6 peroxidatic cysteine (C_P_-SH) is reversibly oxidized to sulfenic acid (C_P_-SOH), which is again reduced to C_P_-SH during the catalytic cycle with the participation of π-glutathione-S-transferase (πGST) and glutathione (GSH) [13,14]. With an increase in the concentration of peroxides, the oxidation of sulfenic acid (C_P_-SOH) can proceed further–to sulfinic (C_P_-SO_2_H) and sulfonic (C_P_-SO_3_H) acids. In this case, the rate of subsequent oxidation of C_P_-SOH to C_P_-SO_2_H can substantially decrease [15]. For instance, the oxidation of human Prdx2 from C_P_-SOH to C_P_-SO_2_H (12 × 10^3^ M^−1^ s^−1^) is almost 1000 times slower than the oxidation of C_P_-SH to C_P_-SOH (20 × 10^6^ M^−1^ s^−1^) [16]. Typical 2-Cys peroxiredoxins (Prdx1-4) have been shown to realize ATP-dependent reduction of C_P_-SO_2_H to C_P_-SOH, which involves sulforedoxins (Srx) and sestrins (SESN) [17,18]. There are no data directly testifying to the reduction of C_P_-SO_2_H for 1-Cys (Prdx6). However, it is known that Srx are not involved in the reduction of C_P_-SO_2_H of Prdx6 [18]. C_P_-SO_3_H is an irreversibly oxidized state for all peroxiredoxins when a complete loss of the peroxidase activity is observed. It must be noted that overoxidized forms (C_P_-SO_2_H/C_P_-SO_3_H) of peroxiredoxins, after the loss of the peroxidase function, begin to play the role of chaperones (2-Cys Prdx), phospholipases (1-Cys Prdx) as well as participate in intracellular and intercellular signaling [19]. 

The phospholipase catalytic center Prx6 is located on the protein surface and includes a triad of amino acids: His26, Ser32, and Asp140 (Figure 1). Mutations in His26 and Ser32 have been shown to result in a loss of both the aiPLA2 activity and the ability of Prx6 to bind phospholipids. Asp140 mutation is attended by a loss of the aiPLA2 activity but does not affect phospholipid binding [20]. It must be noted that despite the spatial remoteness of the Prdx6 catalytic centers (Figure 1), they have a close functional relationship. In particular, it has been demonstrated that the aiPLA2 activity can significantly increase upon oxidation of the peroxidase cysteine residue (Cys47), which occurs at high concentrations of intracellular peroxides [21]. Due to its peroxidase and aiPLA2 activities, Prdx6 ensures the restoration of oxidized phospholipids and the preservation of the normal function of biological membranes under conditions of oxidative stress [7]. Resulting from the phospholipase activity of Prdx6, lysophospholipids and free fatty acids (cleaved off at the 2nd position of triglycerides), which are involved in important cell signaling pathways, are formed from phospholipids. For example, the aiPLA2 of Prdx6 increases the level of lysophosphatidylcholine (LPC), which modulates the activation of a prooxidant enzyme NADPH oxidase type 2 (NOX2) in pulmonary microvascular endothelial cells (PMVECs), alveolar macrophages (AMs), and polymorphonuclear leukocytes [22]. It has been shown that an increase in the aiPLA2 activity in human melanoma cells leads to an increase in the concentration of arachidonic acid, which modulates the activity of Src kinases, thereby stimulating cell division [23]. 

Thus, Prdx6 is a unique enzyme with an essential antioxidant function in the cell [24]. Knockdown or knockout of the *PRDX6* gene leads to a decrease in the cell resistance against the action of oxidative factors (ionizing radiation, cisplatin, etc.) and an increase in cell death [25,26,27]. Contrariwise, *PRDX6* overexpression enhances cell resistance to oxidative stress, stimulates proliferation and favors cell survival [28,29]. *PRDX6* gene knockout animals are highly sensitive to oxidative factors (hyperoxia, action of peroxides, paraquat, etc.), which is attended by an increase in the level of oxidative damage to organs (especially kidneys, liver, and lungs) and tissues of transgenic animals [30]. The important antioxidant function of endogenous Prdx6 prompted us to investigate the protective action of the exogenous form of Prdx6 in various cellular and animal models of oxidative stress [31]. In particular, Prdx6 has proven to be a potent natural radioprotector [32,33]. The radioprotective effect of exogenous Prdx6 is provided not only by its peroxidase activity, but is also due to its signaling and regulatory function mediated by stimulation of the TLR4/NF-κB signaling pathway [26]. It should be noted that the role of the phospholipase activity in the radioprotective effects of Prdx6 was previously unknown. In this study the contribution of phospholipase activity to the radioprotective effect of exogenous Prdx6 was evaluated using an animal model of total irradiation.

It has been recently shown that exogenous Prdx6 efficiently enters eukaryotic cells [26]. We assumed that the aiPLA2 phospholipase activity could play an important role in the transmembrane transfer of Prdx6. In the present study, this hypothesis was verified.

## 2. Results

### 2.1. Characterization of Recombinant Peroxiredoxins

**Figure 2:**
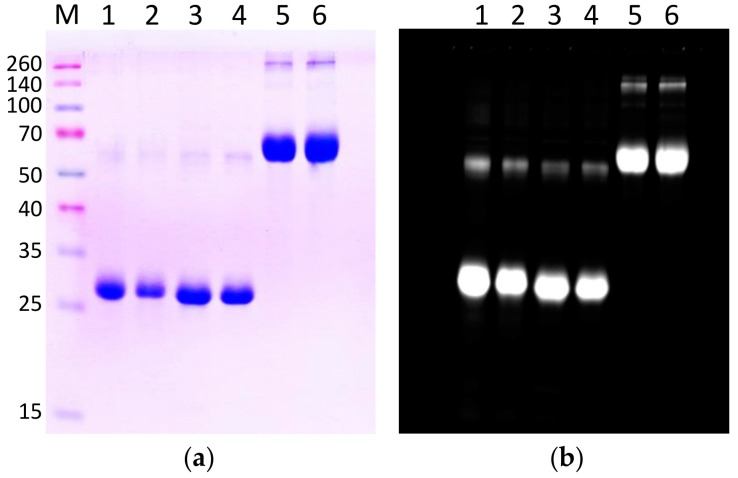
The SDS-PAGE of FITC-labeled proteins used in the study: 1–Prdx6 WT; 2–Prdx6 WT(+); 3–Prdx6-S32A; 4–Prdx6-S32A(+); 5–BSA; 6–BSA(+). (**a**) Coomassie R250 staining; (**b**) UV fluorescence of labeled proteins (iBright, Thermo, USA).

High purity (at least 95%) proteins were used in the work (Figure 2). Evaluation of the peroxidase activity of wild-type (WT) and mutant (S32A) forms of Prdx6 testified that the S32A mutation has no effect on the protein peroxidase activity (Table 1). Prdx6 labeling with FITC resulted in a minor decrease (by 5–10%) of the peroxidase activity of the enzymes. Incubation of Prdx6 in the presence of two mM H_2_O_2_ resulted in irreversible oxidation of the active cysteine residue (Cys47) and a loss of peroxidase activity in both the WT and the S32A mutant (Table 1). Pre-incubation of overoxidized Prdx6(WT/S32A) with five mM DTT did not restore the peroxidase activity, indicating that the peroxidatic cysteine residue (C_P_) is in an irreversibly oxidized state, i.e., predominantly C_P_-SO_2_H/SO_3_H. 

**Figure 3:**
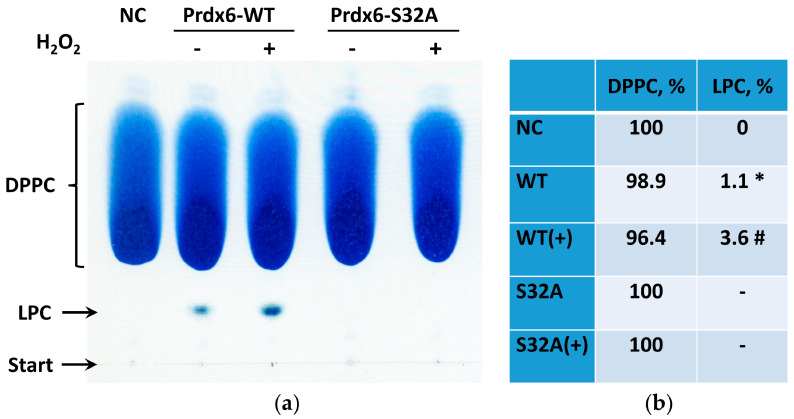
Phospholipase activity of the wild-type form (WT) and deficient for phospholipase active center mutant (S32A) of Prdx6. (**a**) The thin layer chromatography (TLC) of phospholipids following 1-h incubation at pH 5.0 with peroxiredoxins: dipalmitoylphosphatidylcholine (DPPC), lysophosphatidylcholine (LPC). TLC start point is marked on the silica gel plate; (**b**) Results of TLC densitometry (N = 3). * *p* < 0.01–changes are statistically significant relative to the negative control (NC); # *p* < 0.01 changes are statistically significant relative to the group with wild-type Prdx6 protein (WT).

Assessment of the phospholipase activity of Prdx6 enzymes demonstrated that the S32A mutant (both the reduced and overoxidized forms) does not exhibit phospholipase A2 activity (Figure 3). At the same time, overoxidized wild-type protein Prdx6-WT(+) showed an approximately 3-to-4-fold increase in the phospholipase activity (aiPLA2), which correlated with an elevated level of lysophosphatidylcholine (LPC) formed as a result of palmitoyl cleavage at the 2nd position of DPPC (Figure 3).

Upon preparation of sufficient amounts of labeled Prdx6 types differing in phospholipase activity (no activity in S32A, normal in WT, and 3-to-4-fold increase in overoxidized WT(+)), we tested these proteins in cell lines (3T3, RAW 264.7, A549) differing in the level of TLR4 expression (moderate, high, very low/absent), to exclude TLR4-mediated protein transfer into the cell.

### 2.2. Prdx6 (WT and S32A) Transport into 3T3 Cells

**Figure 4:**
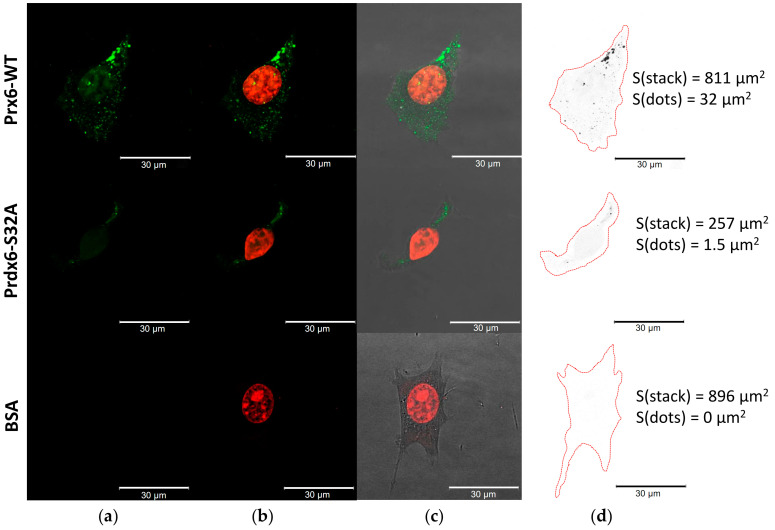
Penetration of exogenous Prdx6 (WT and S32A) into 3T3 cells. (**a**) FAM channel (green) corresponds to FITC–labeled proteins; (**b**) Texas Red channel (red) is aligned with the FAM channel and marks ethidium bromide-stained genomic DNA; (**c**) superimposition of FAM + Texas Red + phase contrast; (**d**) areas analyzed in ImageJ are marked, cell contours are shown in red, stack areas and areas with labeled proteins (dots) are shown at the right. Scale bars are 30 μm.

After the addition of labeled Prdx6-WT to 3T3 mouse embryonic fibroblasts, we registered a significant amount of the labeled protein inside the cells, covering about 3.95% of the confocal stack area (Figure 4, Table 2). 

### 2.3. Prdx6 (WT and S32A) Transport into RAW 264.7 Cells

**Figure 5:**
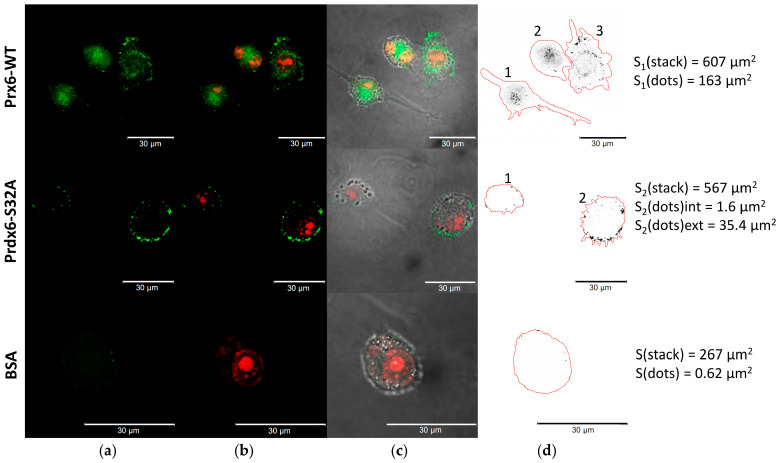
Penetration of exogenous Prdx6 (WT and S32A) into RAW 264.7 cells. (**a**) FAM channel (green) corresponds to FITC-labeled proteins; (**b**) Texas Red channel (red) is aligned with the FAM channel and marks ethidium bromide-stained genomic DNA; (**c**) superimposition of FAM + Texas Red + phase contrast; (**d**) the number of cells in the field of view and areas analyzed in ImageJ are marked, cell contours are shown in red, stack areas and areas with labeled proteins (dots, int–inside the cells, ext–on the cell surface) are shown for the corresponding cells. Scale bars are 30 μm.

Prdx6-WT was shown to effectively penetrate these cells; up to 26.8% of the confocal stack area accounted for the labeled protein. The phospholipase center mutant Prdx6-S32A virtually did not get inside (~0.28%), and most of the labeled protein was concentrated on the cell surface ~6.24% (Figure 5, Table 2). The control BSA-FITC protein was identified in minor amounts in RAW 264.7 cells (0.23%), which is apparently related to their phagocytic activity (Figure 5).

### 2.4. Prdx6 (WT and S32A) Transport into A549 Cells

**Figure 6:**
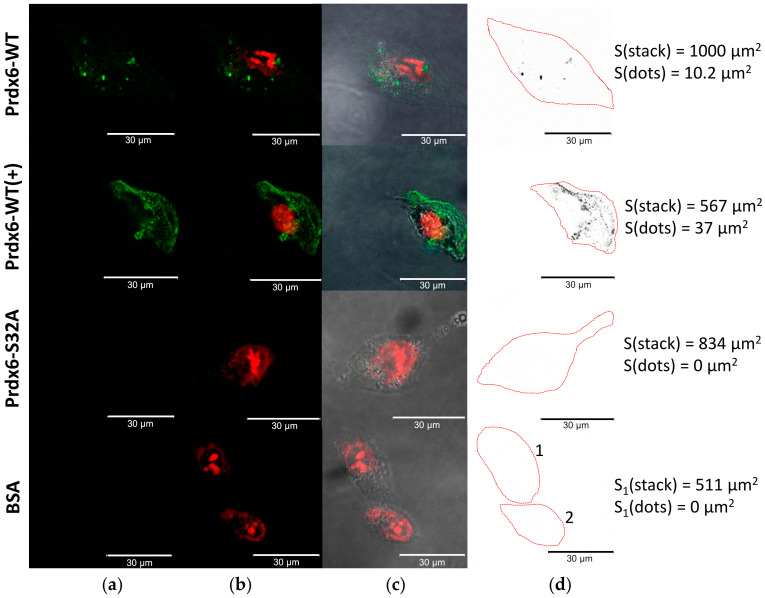
Penetration of exogenous Prdx6 (WT and S32A) into A549 cells. (**a**) FAM channel (green) corresponds to FITC-labeled proteins; (**b**) Texas Red channel (red) is aligned with the FAM channel and marks ethidium bromide-stained genomic DNA; (**c**) superimposition of FAM + Texas Red + phase contrast; (**d**) the number of cells in the field of view and areas analyzed in ImageJ are marked, cell contours are shown in red, stack areas and areas with labeled proteins (dots, int–inside the cells, ext–on the cell surface) are shown for the corresponding cells. Scale bars are 30 μm.

In the case of A549 cells nearly completely lacking TLR4 receptors [34], no protein (both wild-type and mutant) sorption on the cell surface was registered. Notably, only the wild-type protein penetrated the cells, which accounted for approximately 1% of the total stack area. These cells were convenient for evaluation of the ability of exogenous Prdx6 protein for intracellular translocation without the involvement of TLR4. It is known that overoxidation of the peroxidase active center of Prdx6 enhances its phospholipase activity (Figure 2). We evaluated the changes in the transmembrane transport of Prdx6-WT and the Prdx6-S32A mutant overoxidized with 2 mM H_2_O_2_. Irreversibly oxidized Prdx6-WT(+) penetrated into A549 cells nearly 15 times more efficiently (1.02% of the confocal stack area for the initial form versus 14.8% for the oxidized protein), which again indicates the important role of phospholipase activity in cell entry by Prdx6 (Figure 6a, Table 2). At the same time, the Prdx6-S32A mutant (both in reduced and overoxidized forms) was unable to penetrate A549 cells. The control BSA-FITC protein was not detected either on the surface or inside A549 cells (Figure 6). 

### 2.5. Effect of Mutation in the Prdx6 Phospholipase Center on Its Radioprotective Properties

**Figure 7:**
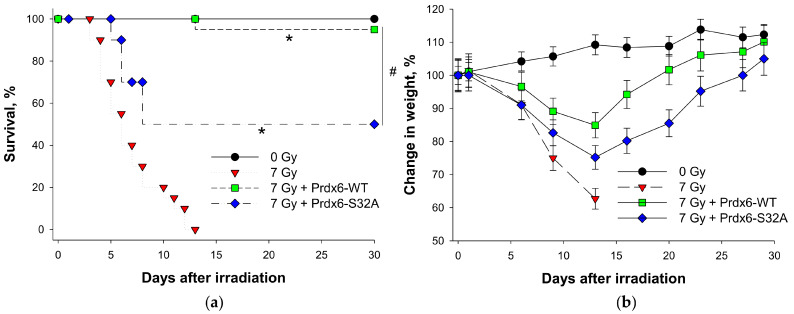
Radioprotective effect of exogenous Prdx6 proteins: wild-type (WT) and mutant for phospholipase center (S32A). Survival (**a**) and changes in the body weight (**b**) of non-irradiated/irradiated animals, not receiving/receiving intravenous administration of recombinant peroxiredoxins (20 μg/g), within 30 days. * *p* < 0.01–significant differences between the experimental groups (Prdx6-WT and Prdx6-S32A) and the irradiated control (7 Gy); # *p* < 0.05–significant differences between the irradiated Prdx6-WT and Prdx6-S32A groups.

We have previously shown that exogenous Prdx6-WT possesses a prominent radioprotective effect [33]. Intravenous administration of mutant Prdx6-S32A protein in kv:SHK mice prior to X-ray irradiation at a lethal dose (7 Gy) caused a significantly lower radioprotective effect in comparison to the initial Prdx6-WT protein, as manifested in a nearly 2-fold reduction of animal survival within 30 days (Figure 7). Moreover, animals injected with Prdx6-S32A before irradiation demonstrated a more rapid weight loss (Figure 7) and consumed lower amounts of feed and water. Hence, with the use of the model of total irradiation of mice with lethal doses of X-rays, it was demonstrated that the phospholipase activity of Prdx6 plays an essential role in its radioprotective action.

## 3. Discussion

Prdx6 is a multifunctional enzyme. In addition to peroxidase activity, Prdx6 exhibits the properties of phospholipase A2 (mainly in acidic environment), which are enhanced by overoxidation of the peroxidase active center (Figure 2) and/or specific phosphorylation of the Thr177 residue [35]. Increased phospholipase activity of Prdx6 results in a raise of the level of lysophospholipids and free fatty acids involved in important cell signaling pathways through modulation of the activity of various protein kinases and transcription factors [36]. For instance, enhanced phospholipase activity of exogenous Prdx6 can favor activation of cell proliferation through stimulation of PI3K/Akt and JAK2/STAT3 signaling pathways [36]. 

When studying the mechanisms of the radioprotective action of Prdx6 on a culture of 3T3 mouse embryonic fibroblasts, a concentration-dependent ability of this protein to penetrate into the cells was identified. [26]. This is apparently the reason why exogenous Prdx6 effectively eliminates intracellular peroxides [26], as well as lowers the level of the redox-sensitive transcription factor p53, which promotes a suppression of senescence and apoptosis of irradiated cells [37]. Apart from this, we have recently shown that overexpression of exogenous Prdx6 in the cells also causes p53 suppression [38]. 

**Figure 8:**
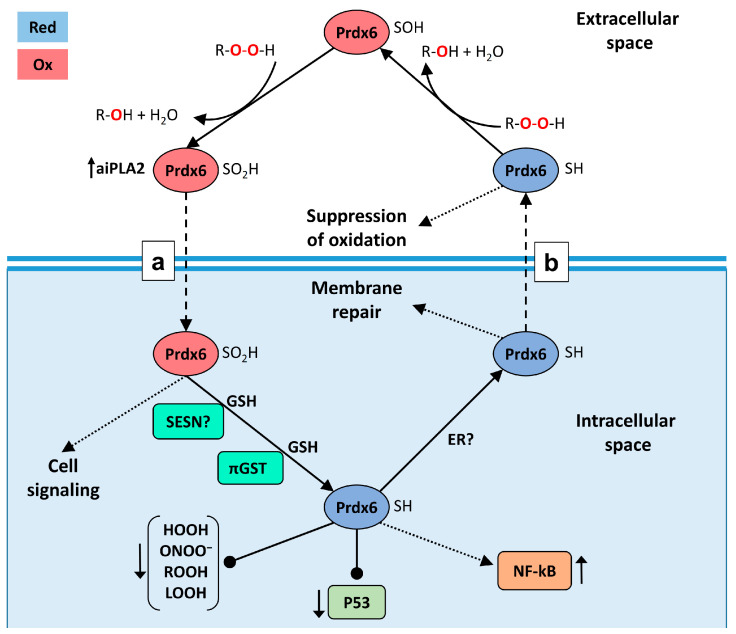
A hypothetical chart of Prdx6 “recirculation” between the cell and the extracellular space, and the role of the phospholipase activity (aiPLA2) in this process. (**a**) Penetration of Prdx6 into the cell; (**b**) secretion of Prdx6 into the extracellular space. The oxidized form of Prdx6 can influence intracellular processes and, after going through reduction steps, can be involved again in antioxidant defense. Glutathione (GSH), sestrins (SESN), and/or others redox-enzymes are involved in the reduction of the oxidized Prdx6-SO_2_H form. Prdx6-SOH is being reduced with the participation of GSH and glutathione-S-transferase π (πGST). The reduced form of Prdx6 is involved in the suppression of cell membrane oxidation and the reduction of oxidized phospholipids. The reduced (Red) form of Prdx6 is shown in blue, the oxidized (Ox) form is in red. ER–endoplasmic reticulum. Dotted arrows denote an indirect action. Normal arrows denote direct actions. Arrows with a bold dot denote inhibition/suppression.

We suggested that the penetrability of Prdx6 may be related to its phospholipase activity. For verification of this hypothesis, three forms of Prdx6 were tested: the original protein (WT), protein with increased phospholipase activity due to overoxidation of the peroxidase center (WT+), and a mutant (S32A) lacking phospholipase activity. Since Prdx6 is a ligand for the TLR4 receptor [26], its sorption on the cell surface with subsequent internalization of the TLR4/Prdx6 complex is highly probable. Therefore, it was necessary to distinguish the specific intracellular penetration of the labeled exogenous Prdx6 from the transmembrane transfer as part of the TLR4/Prdx6 complex. For this purpose, experiments were carried out in three cell lines (3T3, RAW 264.7, A549) having different amounts of TLR4 on the surface [34,39,40]. Tests conducted in these cell cultures witnessed a sharp reduction in the transport of exogenous Prdx6 into the cells upon inactivation of the phospholipase active center in Prdx6-S32A (Table 2). The results obtained in RAW 264.7 cells are the most conclusive (Figure 4). The mutant Prdx6-S32A form (following 1.5-h incubation) was abundantly concentrated on the cell surface, apparently in a complex with TLR4. At the same time, an extremely small portion of the Prdx6-S32A protein (probably as part of complex with TLR4) penetrated into RAW 264.7 cells. The A549 culture, which has virtually no TLR4 receptors, did not demonstrate the binding of Prdx6 (both the original protein and the mutant) to the cell surface. Importantly, only the protein with normal phospholipase center penetrated into A549 cells (Figure 5). It is of interest to note that overoxidation of the Prdx6 peroxidase center, leading to an approximately 2-to-3-fold increase in the phospholipase activity (Figure 2), resulted in a nearly 15-fold enhanced penetrability of exogenous Prdx6-WT (Figure 5, Table 2). This finding is yet another confirmation of the tight functional relationship between the phospholipase and peroxidase activities of Prdx6 [7]. It should be also mentioned that oxidation with two mM H_2_O_2_ promotes an increase in the formation of dimers and oligomers in Prdx6 solution [41,42], which can also affect the penetrability of the protein. The results obtained in A549 lung adenocarcinoma cells provide a novel insight into the function of Prdx6 in the respiratory system tissues. Prdx6 is secreted into the mucosa along the respiratory tract (olfactory epithelium, trachea, bronchi, and alveolar mucosa), ensuring antioxidant protection of the lung tissue and normal surfactant metabolism [2,8]. Activation of aiPLA2 during Prdx6 oxidation can promote its entry into the cell where it can be reduced to the active state [43]. Intracellular glutathione-S-transferase π (πGST) is known to be a natural reducer of Prdx6 [44], capable of restoring the oxidized peroxidase cysteine residue (Cys47-SOH) to its active state (Cys47-SH) [13]. Liposomal delivery of oxidized Prdx6 (C_P_-SOH) to H441 cells, which do not express Prdx6 and express πGST, has been previously shown to increase the peroxidase activity of these cells [45]. Apart from that, it has been testified in PMVEC mouse lung endothelial cells that only the oxidized form of Prdx6 interacts with πGST [46]. At the same time, other intracellular enzymes, such as sestrins (SESN) or other redox-active enzymes, can also participate in the reduction of oxidized Prdx6 (-SO_2_H) [17,47]. Thus, the phospholipase activity may be involved in the recovery and some kind of “recirculation” of secretory/exogenous Prdx6, i.e., the enzyme enters the cell in the oxidized form, being again secreted into the intercellular space in the reduced form (Figure 8). 

It is also important to mention that the sequence between amino acids 31 and 40 (DSWGILFSHP) in the N-terminal region of Prdx6 plays an important role in targeting the protein from the endoplasmic reticulum (ER) to lamellar bodies, which secrete it into the extracellular space. Moreover, substitution of serine 32 for alanine (S32A) abolished the organellar localization of Prdx6 in human (A549) and mouse (MLE12) lung cells [48]. In addition, Prdx6 translocation into mitochondria has been demonstrated under conditions of oxidative stress, at the initial stage of PINK1/Parkin-mediated mitophagy [49]. It is possible that it is namely the phospholipase activity that plays an important role in the processes of Prdx6 translocation both into lamellar bodies and mitochondria. 

It should be mentioned that we have demonstrated earlier a powerful cytoprotective effect of exogenous Prdx6, using models of chemical and thermal burns of the upper respiratory tract in rats, which was manifested in a significant preservation (by 50% higher compared to the control) of the ciliated epithelium of the trachea [50]. This can be explained not only by the elimination of a wide range of hydroperoxides in the damaged area, but also, obviously, by the ability of Prdx6 to be effectively reduced and re-engaged (due to aiPLA2) in the antioxidant defense of epithelial cells. The important role of the aiPLA2 activity of the extracellular/secretory form Prdx6 in cell membrane remodeling, which may also be of critical importance in the repair of damaged tissues, also cannot be excluded. 

Similar results have been obtained for exogenous Prdx6 in other models of free-radical pathologies [31,51]. In particular, we have shown that exogenous Prdx6 exerts a radioprotective effect in model experiments with cell cultures and animals. Notably, inactivation of the peroxidase active center (Prdx6-C47S mutant form) reduced the radioprotective effect by approximately 80% [26,33,52]. The role of the phospholipase activity (aiPLA2) of exogenous Prdx6 in its radioprotective effect remained vague until now. Experiments with the Prdx6-S32A mutant form (with inactivated aiPLA2 and normal peroxidase activity) in a model of total animal irradiation with a lethal dose (7 Gy) of X-rays exhibited an almost two-fold decrease in the efficiency of protection (Figure 7a), indicating the important role of aiPLA2 in the radioprotective effect of Prdx6. Apparently, the weakening of the radioprotective effect of Prdx6-S32A (despite the presence of a normal peroxidase center) may be due to the suppression of its transmembrane transport into the cells. This in turn reduces the efficiency of elimination of intracellular ROS (which have a crucial role in cell death), as well as disables the reduction of exogenous Prdx6 and its subsequent secretion in the active state (Figure 8). 

Perhaps it is due to the phospholipase activity that exogenous Prdx6 not only directly affects the level of intracellular ROS, but also takes part in the redox relay regulation of the most important transcription factors [3,26,37], thus influencing the fate of the cell (Figure 8). Additionally, exogenous Prdx6 activates NF-κB expression through TLR4 stimulation [26] and inhibits p53 activity [37], which promotes cell survival under conditions of oxidative stress caused by ionizing radiation. It has been recently shown that Prdx6 is capable of forming cation-selective ion channels in artificial membranes [53], which are selective to Ca^2+^ [54]. Calcium ions are known to be involved in both secretion of proteins [55] and endocytosis of TLR4/LPS complex [56]. Hence, Ca^2+^-selective ion channels of Prdx6 can complement the aiPLA2 activity in the process of its transport into the cell and/or secretion. These assumptions clearly require further detailed investigations; however, it is no doubt that Prdx6 is a critically important antioxidant enzyme realizing multiple functions in the cell, which are far from being completely understood.

## 4. Materials and Methods

### 4.1. Genetically Engineered Constructs

The procedure of human PRDX6 gene cloning was described in our previous study in detail [33]. The obtainment of the mutant form with inactivated phospholipase activity (PRDX6-S32A) was conducted in accordance with the approach based on the use of overlapping primers, with Tersus high-fidelity DNA polymerase (Evrogen, Moscow, Russia), oligonucleotides ensuring a single-nucleotide substitution (S32A-F: 5’-CTGGGAGACGCATGGGGCAT-3’; S32A-R: 5’-ATGCCCCATGCGTCTCCCAG-3’) and flanking primers (PRDX6-F: 5’-TTTTTCATATGCCCGGAGGTCTGCTT-3’, with NdeI restriction site), PRDX6-R: 5’-AATTCTCGAGAGGCTGGGGTGTGTA-3’, with XhoI restriction site). The PRDX6 gene with the S32A substitution was cloned into a bacterial expression plasmid pET23b by NdeI and XhoI restriction sites. The accuracy of genetically engineered construct (pET23b-PRDX6-S32A) was confirmed by sequencing. 

### 4.2. Purification of Recombinant Proteins

The C-terminus of Prdx6 proteins (WT/S32A) contains six histidine residues (His-tag), which allows for their purification on Ni-NTA agarose (Invitrogen, Carlsbad, CA, USA) under native conditions. The procedure of purification of recombinant peroxiredoxins from *Escherichia coli* BL21(DE3) bacterial culture was described in detail in one of our earlier works [57]. According to electrophoresis in sodium dodecyl sulfate-12.5% polyacrylamide gel (SDS-PAGE), the purity of the obtained proteins was at least 95%. The level of LPS in recombinant proteins was estimated using LAL test (Sigma-Aldrich, Saint Louis, MO, USA) according to the manufacturer’s instructions. LPS levels in Prdx6 (WT/S32A) preparations were less than 2 ng/1 mg protein. Residual *E. coli* genomic DNA in recombinant Prdx6 (WT/S32A) was determined using real-time PCR, as described previously [58], and was less than 10 ng/1 mg of protein. The obtained proteins were filtered using PTFE syringe filters (Teknokroma, Barcelona, Spain) with 0.22 μm pores, stored at −20 °C before use. No changes in the enzymatic activity of protein were detected during their storage. 

### 4.3. Evaluation of Peroxidase Activity

The peroxidase activity of proteins relative to H_2_O_2_ and tert-butyl hydroperoxide (t-BOOH) was determined by a colorimetric method based on the reaction of oxidation of Fe(II) to Fe(III) with residual peroxide; the concentration of the latter was determinedthrough the reaction with KSCN. The method for determining peroxidase activity is described in detail in [57]. 

### 4.4. Evaluation of Phospholipase Activity

Liposomes were prepared from 18 mM dipalmitoylphosphatidylcholine–DPPC (Avanti Polar Lipids, Alabaster, AL, USA) in acetate buffer (40 mM CH_3_COONa, pH 5.0, 10 mM EDTA) by extrusion through 100 nm pores of a Nuclepore membrane (Whatman, Maidstone, UK). According to dynamic light scattering (DLS), the size range of liposomes was 100 ± 20 nm. To a volume of 200 µL of the resulting liposome suspension, 100 µL of wild type (WT) or mutant (S32A) form Prdx6 protein solution (1 mg/mL), as well as their overoxidized forms (+) was added. Incubation was carried out at +37 °C for 1 h. Subsequently, lipids were extracted with 1 mL of methanol:chloroform (1:2) mixture. The phases were separated by centrifugation, and the chlorophorm (lower) phase with lipids was collected. Chloroform was vacuum-evaporated at +40 °C, and dried lipids were dissolved in 100 μL of methanol:chloroform (1:1) mixture, with 10 μL being taken for application to a silica gel plate and subjected to thin layer chromatography (TLC). Ascending TLC was carried out on a silica gel plate (Merck, Darmstadt, Germany) in a chamber with a solution of chloroform:methanol:water (65:25:5). Then the plate was dried and phospholipids were stained with molybdenum blue according to the method of Vaskovsky [59]. Phospholipase activity was determined by the formation of lysophosphatidylcholine (LPC). The changes in the level of LPC were assessed by TLC with densitometric analysis using ImageJ v.1.8.0 software (National Institutes of Health, Bethesda, MD, USA).

### 4.5. Preparation of Overoxidized Forms of Proteins

To obtain irreversibly oxidized forms of proteins, which are marked with (+), 20 mg of corresponding Prdx6 (WT/S32A) were incubated in 2 mL of 1xPBS with 2 mM H_2_O_2_ for 1 h at +37 °C. The proteins were then dialyzed (in dialysis bags with a molecular weight cut-off of 3.5 kDa) against 1xPBS until completely removal of H_2_O_2_. The proteins were concentrated (to ~20 mg/mL) using Spin-X UF 20 centrifuge concentrators (Corning, Glendale, AZ, USA), with a molecular weight cut-off of 10 kDa. Irreversible oxidation of the Prdx6 (WT/S32A) peroxidase center was confirmed by assessing the peroxidase activity of the enzymes. BSA, used as a negative control, was treated similarly. The obtained proteins were filtered through PTFE syringe filters (Teknokroma, Barcelona, Spain) with 0.22 μm pores and stored at −20 °C until use.

### 4.6. Preparation of FITC-Labeled Proteins

Prdx6 proteins (WT/S32A) were labeled with FITC (Reachim, Moscow, Russia). Briefly, 2 mg FITC in DMSO (50 μL) was added to 10 mg Prdx6 (WT/S32A) in 100 mM carbonate buffer (NaHCO_3_/Na_2_CO_3_), pH 9.0 (450 μL). The mixture was incubated for 1.5 h at +37 °C with agitation. Then, the Prdx6-FITC conjugate was purified from unbound FITC by gel filtration (Sephacryl S-200, Amersham, Marlborough, MA, USA). The labeled protein was concentrated using a centrifugal concentrator Spin-X UF 20 (Corning, USA), with a molecular weight cut-off of 10 kDa, and additionally dialyzed against 1xPBS (~100 volumes of the original protein solution) [57]. FITC-labeled BSA (Amresco, Solon, OH, USA), used as a control, was obtained in a similar way. The protein:FITC molar ratio was ~1:2 (according to the ratio of absorption at 280 nm and 485 nm). The resulting proteins were filtered through PTFE syringe filters with 0.22 μm pores and stored at −20 °C until use.

### 4.7. Cell Cultures

Since Prdx6 is a TLR4 ligand [26], it can penetrate into the cell in the form of a complex (TLR4/Prdx6). Therefore, it was necessary to test exogenous Prdx6 (WT and S32A) in cell lines that differ in the level of TLR4 expression. For that purpose, we used 3T3 mouse embryonic fibroblast cells with an average level of TLR4 [39], RAW 264.7 mouse macrophage cells highly expressing TLR4 [40], and A549 human lung carcinoma cells which have nearly no TLR4 expression (which results in their low sensitivity to lipopolysaccharides) [34]. Cells were seeded in culture flasks (25 cm^2^) to a density of 1 × 10^6^ cells/flask in DMEM medium (Paneco, Moscow, Russia), supplemented with 10% fetal bovine serum–FBS (Thermo Fisher Scientific, Swindon, UK) and a mixture of antibiotics/antimycotics (Sigma- Aldrich, USA). Cell culturing was carried out at 37 °C with 5% CO_2_. Passage 5–8 cells were used in the study. Cell cultures used in this work were regularly tested for mycoplasma infections (Figures S1–S3): using microscopic control, Hoechst 33342 staining (500 ng/mL) (Thermo Fisher Scientific, Eugene, OR, USA) [60] and using real-time PCR [61].

### 4.8. Microscopy

Before the addition of labeled proteins, the cells were washed with DMEM medium from the nutrient medium containing 10% FBS so as to exclude the effect of serum proteins on the experiment. Selection of the optimal concentration of FITC-labeled protein in cell culture is presented in the Supplementary Material (Figure S4). FITC-labeled proteins (Prdx6-WT, Prdx6-S32A and BSA) were added at a final concentration of 0.1 mg/mL to the respective cell cultures (~10^3^ cells/well) in DMEM medium. Following incubation (1.5 h) with proteins, the cells were washed thrice with 1xPBS, fixed with 4% formaldehyde solution (30 min) and permeabilized with 0.2% Triton X-100 (10 min). After that, a solution of ethidium bromide (1 mg/mL) was added to fixed, permeabilized cells, which were then incubated for 5 m and washed again three times with 1xPBS. Microscopic analysis of cell cultures was performed using a Leica TCS SP-5 microscope, Leica Application Suite X software (Leica Microsystems CMS GmbH, Wetzlar, Germany) and ImageJ v.1.8.0 software (National Institutes of Health, Bethesda, MD, USA). In each experimental group, at least five cells were examined, for each of which at least 12 confocal sections (stacks, z-stack) with a step of 1 μm were obtained. A confocal section corresponding to the geometric center of the cell was used for intergroup comparison.

### 4.9. Animals

Male mice of the outbred Kv:SHK line aged 5–6 weeks and weighing 23–26 g (vivarium of ICB RAS) were used in the investigation. Animals were quarantined for 2 weeks prior to the beginning of the experiments. Only healthy animals were used for the study. The animals were kept in the vivarium of ICB RAS at a temperature of 22 °C and 12/12 h light-dark cycles. The animals were given tap water and a balanced feed for mice (Arno, Moscow, Russia) *ad libitum* throughout the experiment. Experiments with animals were carried out in compliance with bioethical norms, in full accordance with the methodological guidelines of ICB RAS for handling laboratory animals No. 57 (30 December 2011), ethical protocol No. 2019/5.

### 4.10. Irradiation of Animals

Animals were irradiated using a RUT-15 X-ray apparatus (Mosrentgen, Moscow, Russia) at a dose rate of 1 Gy/min (current–20 mA, voltage–200 kV, focal length–37.5 cm, solid angle–30°). Total irradiation of mice was carried out at a dose of 7 Gy at room temperature. Each experimental group (control, administration of Prdx6-WT or Prdx6-S32A), contained 30 animals. Intravenous administration of protein solutions (at a dose of 20 μg/g) was implemented 15 min prior to irradiation.

### 4.11. Statistical Analysis

Statistical analysis was performed in SigmaPlot 11.0 (Systat Software Inc., San Jose, CA, USA). The results were expressed as mean ± standard deviation (SD). Inter-group statistical differences were determined using one-way ANOVA analysis, and statistical significance between individual experimental groups was calculated with unpaired Student’s *t*-test. Data with *p* < 0.05 were considered statistically significant.

## 5. Conclusions

The phospholipase activity of Prdx6 ensures the penetration of the exogenous protein into mammalian cells. Overoxidation of the Prdx6 peroxidase center increases the aPLA2 phospholipase activity and favors more efficient penetration of the protein into the cell. Inactivation of the phospholipase active center through introduction of the S32A mutation completely suppresses the penetrating ability of Prdx6. aiPLA2 activity has an important role in the radioprotective action of exogenous Prdx6. The mutant form Prdx6-S32A has a 50% less effective radioprotective action compared to the normal protein. We suggest that aiPLA2 may be of high importance in the intracellular signaling of exogenous Prdx6, as well as in its recovery inside the cell and re-secretion into the intercellular space (Figure 8). These assumptions require further detailed investigation.

## Figures and Tables

**Table 1:** Peroxidase activity of Prdx6: wild-type (WT) and mutant (S32A) forms labeled with FITC and overoxidized with 2 mM H_2_O_2_ (+).

Prdx6	H_2_O_2_, nmol/min/mg	tBOOH, nmol/min/mg
WT	200 ± 10	100 ± 5
WT(+)	10 ± 5	5 ± 2
WT-FITC	185 ± 5	98 ± 5
WT-FITC(+)	8 ± 4	5 ± 2
S32A	200 ± 10	95 ± 5
S32A(+)	9 ± 4	4 ± 2
S32A-FITC	180 ± 10	92 ± 5
S32A-FITC(+)	10 ± 4	4 ± 2

**Table 2:** Penetration of exogenous labeled Prdx6(WT and S32A) and BSA proteins into the cells.

FITC-Labeled Proteins	Intracellular Contents of Exogenous Protein, % from the Total Area of Confocal Stack		
	3T3	RAW 264.7	A549
BSA	0.0	0.23 ± 0.12	0.0
Prx6-WT	3.95 ± 0.35	26.8 ± 1.89	1.02 ± 0.15
Prx6-WT(+)	N/D	N/D	14.8 ± 0.71 **
Prx6-S32A	0.56 ± 0.10 *	0.28 ± 0.12 **	0.0
Prx6-S32A(+)	N/D	N/D	0.0

N = 5 in each group. The values are statistically significant (* *p* < 0.01, ** *p* < 0.001) in comparison to the Prdx6-WT group. N/D—no data.

## Data Availability

All data are included in the article and Supplementary Materials.

## Supplementary Materials

The following supporting information can be downloaded at: https://www.mdpi.com/article/10.3390/ijms232315265/s1.
