# Supplementary material for: The Role of Phospholipase Activity of Peroxiredoxin 6 in Its Transmembrane Transport and Protective Properties
Source: Int J Mol Sci. 2022 Dec 3;23(23):15265. doi: 10.3390/ijms232315265 (PMC9738660; doi:10.3390/ijms232315265)
Supplement: Supplementary file 1 [file ijms-23-15265-s001.zip › ijms-2039030-supplementary.pdf]

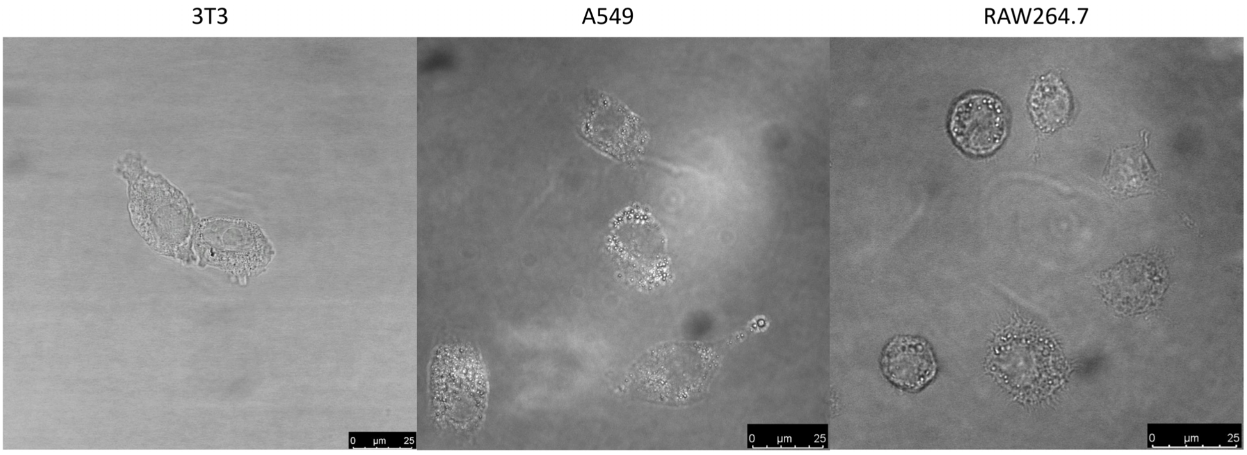

Figure S1. Phase-contrast microscopy of the cells used in the study. The cells have normal morphology for their type, no mycoplasmas (up to 1.5 µm in size) were detected. Scale bars are 25 µm.

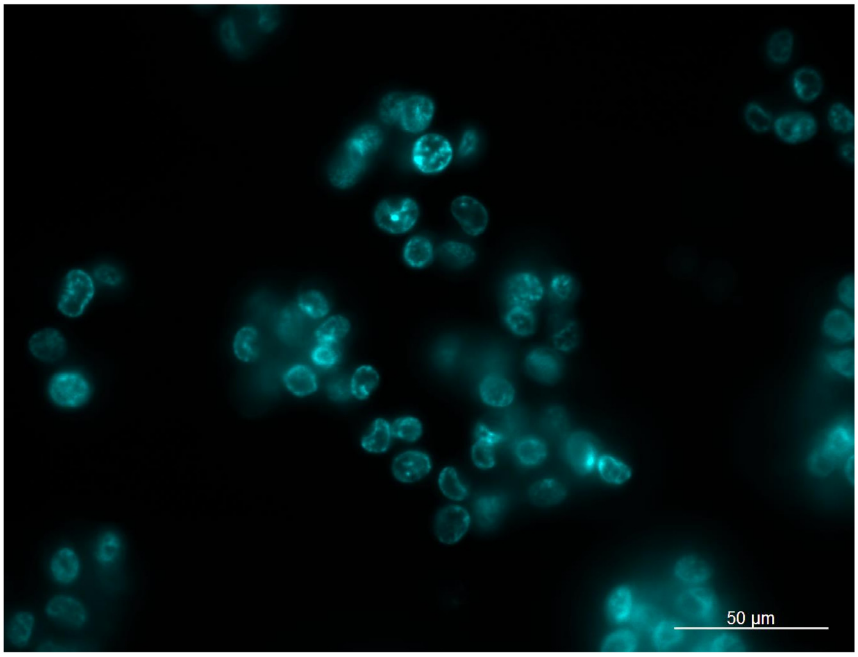

Figure S2. DNA staining of 3T3 cells with Hoechst 33342 dye. Staining of nuclear DNA of 3T3 cells (objects 10-15 µm in size) was observed. Mycoplasma DNA (objects up to 1.5 µm in size) was not detected. Scale bar 50 µm.

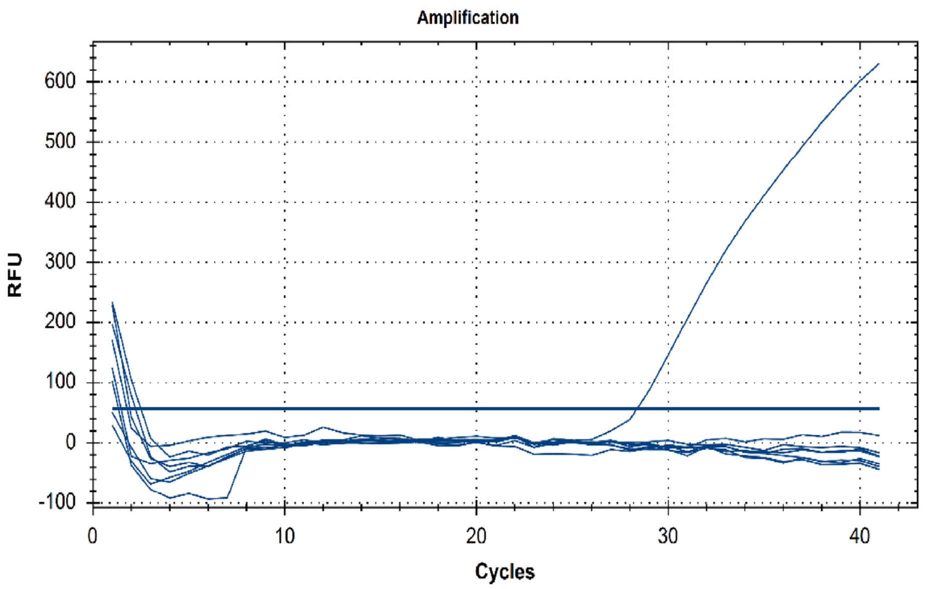

Well	Fluor	Target	Sample	Ct
A01	FAM	Mycoplasma spp	3T3_1	-
A02	FAM	Mycoplasma spp	3T3_2	-
A03	FAM	Mycoplasma spp	RAW264.7_1	-
A04	FAM	Mycoplasma spp	RAW264.7_2	-
A05	FAM	Mycoplasma spp	A549_1	-
A06	FAM	Mycoplasma spp	A549_2	-
A07	FAM	Mycoplasma spp	NTC	-
A08	FAM	Mycoplasma spp	PC	28,06

Figure S3. Real-time PCR results for 16S rDNA of *Mycoplasma spp*. PCR was performed according to the method [Janetzko et al., 2014], with a modification of the detection probe: FAM-TGGTGCATGGTGGTTGTCGTCAGCTCTGTCGT-BHQ. DNA isolated from "AVIVAK RM" vaccine containing attenuated culture of *Mycoplasma gallisepticum* (AVIVAK Research and Production Enterprise, Russia) was used as a control. Genomic DNA isolation from cell cultures and positive controls was performed using LumiPure from AnySample (Lumiprobe, Russia). Mycoplasma DNA was not detected in the studied cell cultures (3T3, A549, RAW264.7).

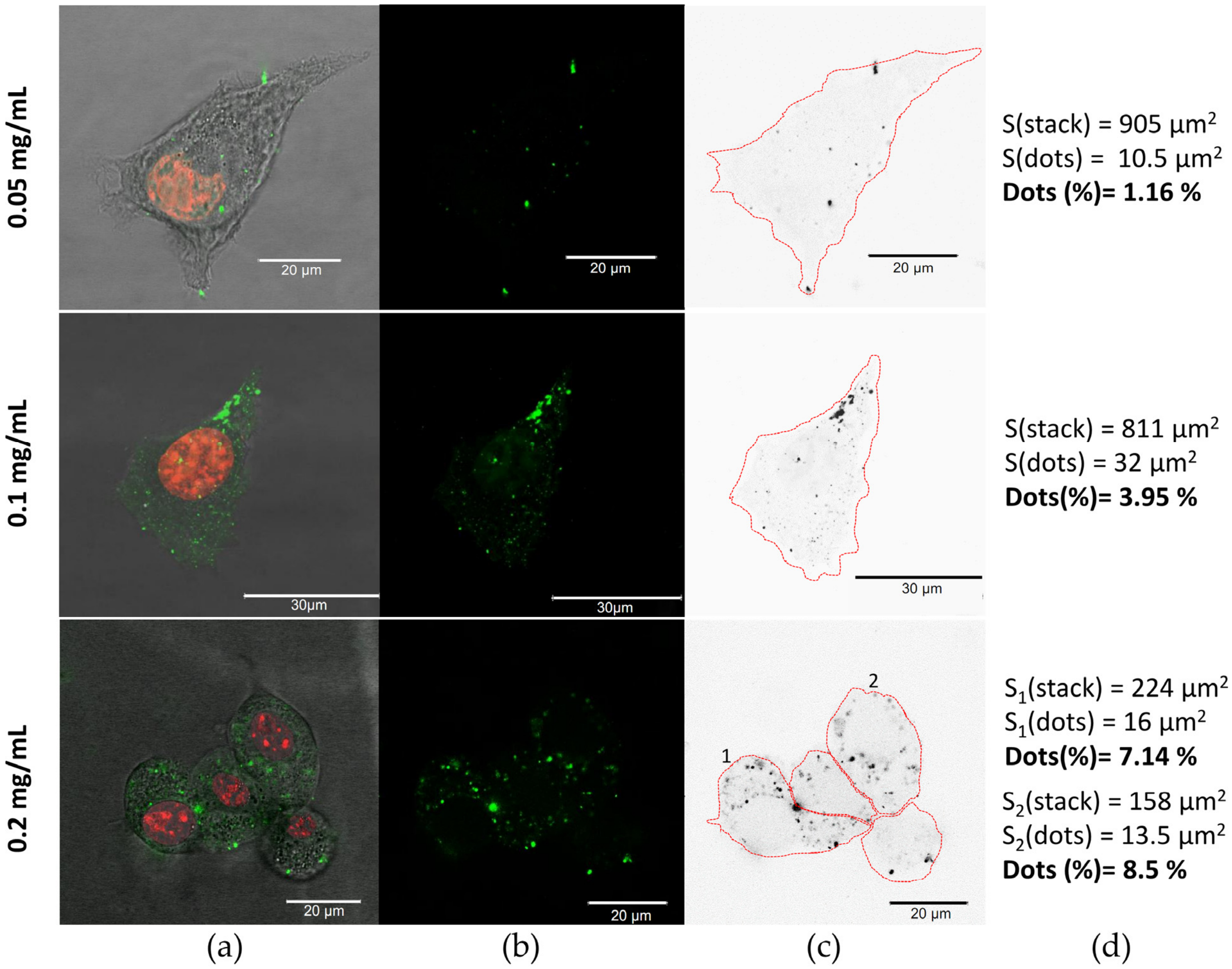

Figure S4. Concentration dependence of the penetration of exogenous Prdx6-WT (FITC-labeled) into 3T3 cells. (**a**) superimposition of FAM + Texas Red + phase contrast; (**b**) FAM channel (green) corresponds to FITC-labeled Prdx6 at different concentrations (0.05, 0.1, 0.2 mg/mL); (**c**) areas analyzed in ImageJ are marked, cell contours are shown in red; (**d**) assessment of labeled protein content in cells. Scale bars are 20 and 30 µm.
